# High-fat diet-induced obesity exacerbates kainic acid-induced hippocampal cell death

**DOI:** 10.1186/s12868-015-0202-2

**Published:** 2015-10-30

**Authors:** Dong Ho Kang, Rok Won Heo, Chin-ok Yi, Hwajin Kim, Chang Hwa Choi, Gu Seob Roh

**Affiliations:** Department of Neurosurgery, Institute of Health Sciences, Gyeongsang National University School of Medicine, 15, Jinju-daero 816 Beon-gil, Jinju-si, Gyeongnam Republic of Korea; Department of Anatomy and Convergence Medical Science, Institute of Health Sciences, Gyeongsang National University School of Medicine, 15, Jinju-daero 816 Beon-gil, Jinju-si, Gyeongnam Republic of Korea; Department of Neurosurgery, Pusan National University School of Medicine, 179 Gudeok-ro, Seo-gu, Busan, Republic of Korea

**Keywords:** Obesity, Excitotoxicity, Neuroinflammation, Oxidative stress, Hippocampus

## Abstract

**Background:**

Obesity has deleterious effects on the brain, and metabolic dysfunction may exacerbate the outcomes of seizures and brain injuries. However, it is unclear whether obesity affects excitotoxicity-induced neuronal cell death. The purpose of this study was to investigate the effects of a high-fat diet (HFD) on neuroinflammation and oxidative stress in the hippocampus of kainic acid (KA)-treated mice.

**Results:**

Mice were fed with a HFD or normal diet for 8 weeks and then received a systemic injection of KA. HFD-fed mice showed hypercholesterolemia, insulin resistance, and hepatic steatosis. HFD-fed mice showed greater susceptibility to KA-induced seizures, an increased number of terminal deoxynucleotidyl transferase dUTP nick end labeling (TUNEL)-positive cells, neuroinflammation, and oxidative stress. Furthermore, we found that KA treatment increased HFD-induced calpain1, nuclear factor E2-related factor 2, and heme oxygenase-1 expression in the hippocampus.

**Conclusions:**

These findings imply that complex mechanisms affected by obesity-induced systemic inflammation, neuroinflammation, ER stress, calcium overload, and oxidative stress may contribute to neuronal death after brain injury.

**Electronic supplementary material:**

The online version of this article (doi:10.1186/s12868-015-0202-2) contains supplementary material, which is available to authorized users.

## Background

Patients with epilepsy have a greater risk for obesity than the general population due to treatment of anti-epileptic medications [1, 2], and obesity is also a common comorbidity in children with untreated epilepsy [3, 4]. Although metabolic dysfunction is known to modulate the outcome of brain injury and increase seizure severity [5], it remains unclear the molecular mechanisms that metabolic dysfunction causes excitotoxicity-induced neuronal death. However, accumulating evidence suggests that metabolic dysfunction could have a deleterious effect on excitotoxicity-induced neuronal death. Specifically, a high-fat diet (HFD) is associated with brain insulin resistance and compromised synaptic integrity [6]. Adiponectin-deficient mice exhibit insulin resistance, hyperlipidemia, and inflammation [5, 7], and adiponectin-deficient mice fed a HFD display increased seizure severity, whereas injection of adiponectin reduces kainic acid (KA)-induced excitotoxicity [5, 8]. Furthermore, the neurotoxins methamphetamine and KA increase mortality and neurodegeneration in ob/ob mice more than in lean mice [9].

KA-induced hippocampal changes are associated with blood-brain barrier (BBB) leakage, astrogliosis, inflammation, oxidative stress, calcium overload, apoptosis, and endoplasmic reticulum (ER) stress [10–13]. Nuclear factor-kappaB (NF-κB)-mediated neuroinflammation is induced by seizures and increases susceptibility to excitotoxicity-induced neuronal cell death [14]. In particular, the nuclear factor E2-related factor 2 (Nrf2)-mediated defense pathway is involved in neuronal sensitivity to KA [15], and activation of this pathway exerts neuroprotective effects by elevating antioxidant enzymes [16]. For example, Nrf2 plays an important role in protecting hepatocytes from hepatic steatosis in HFD-fed mice [17]. Obesity-induced mitochondrial dysfunction and oxidative stress also contribute to the initiation and progression of seizures [9]. In our previous study, we showed that an ER stress inhibitor attenuates KA-induced hippocampal cell death [13]. However, to our knowledge, whether the Nrf2-mediated antioxidant pathway plays a role in KA-induced brain injury in HFD-fed mice has not been studied.

Therefore, the aim of the present study was to determine whether chronic HFD-induced obesity contributes to deleterious effects of KA treatment on the hippocampus and to investigate several mechanisms including inflammation and oxidative stress between metabolic dysfunction and neuronal injury. We also examined whether obesity-induced systemic inflammation exacerbates the inflammatory changes induced by KA treatment.

## Results

### Effect of a HFD on obesity-related phenotypes and insulin resistance

Mice were fed with a normal diet (ND) or HFD for 8 weeks. HFD mice had heavier body weights, livers and epididymal fat pads than ND mice (Additional file 1: Fig. S1A–D). H&E staining revealed that HFD mice had distended hepatocytes with accumulated fat and epididymal fat pads with the presence of many macrophages (Additional file 1: Fig. S1E). HFD mice also had higher levels of hepatic triglyceride (TG) than ND mice (Additional file 1: Fig. S1F). To confirm whether a HFD causes metabolic dysfunction, we measured levels of serum lipid metabolic factors and hepatic enzymes. HFD mice displayed elevated levels of serum free fatty acid (FFA), aspartate aminotransferase (AST), alanine aminotransferase (ALT), total cholesterol, and TG (Additional file 2: Table S1).

To confirm the effect of a HFD on insulin resistance, we measured blood glucose and serum insulin level, and performed glucose tolerance test (GTT) and insulin tolerance test (ITT) (Additional file 3: Fig. S2A–D). After 4 weeks, HFD mice were more glucose tolerant than ND mice (Additional file 3: Fig. S2A). HFD mice showed hyperinsulinemia and a greater increase in glucose levels than ND mice during an ITT and a GTT (Additional file 3: Fig. S2B–D). HFD mice also exhibited hypoadiponectinemia (Additional file 3: Fig. S2E) and increased serum levels of interleukin-6 (IL-6) and tumor necrosis factor (TNF)-α compared with ND mice (Additional file 3: Fig. S2F, G).

### Effect of a HFD on KA-induced seizure activity and mortality

**Fig. 1:**
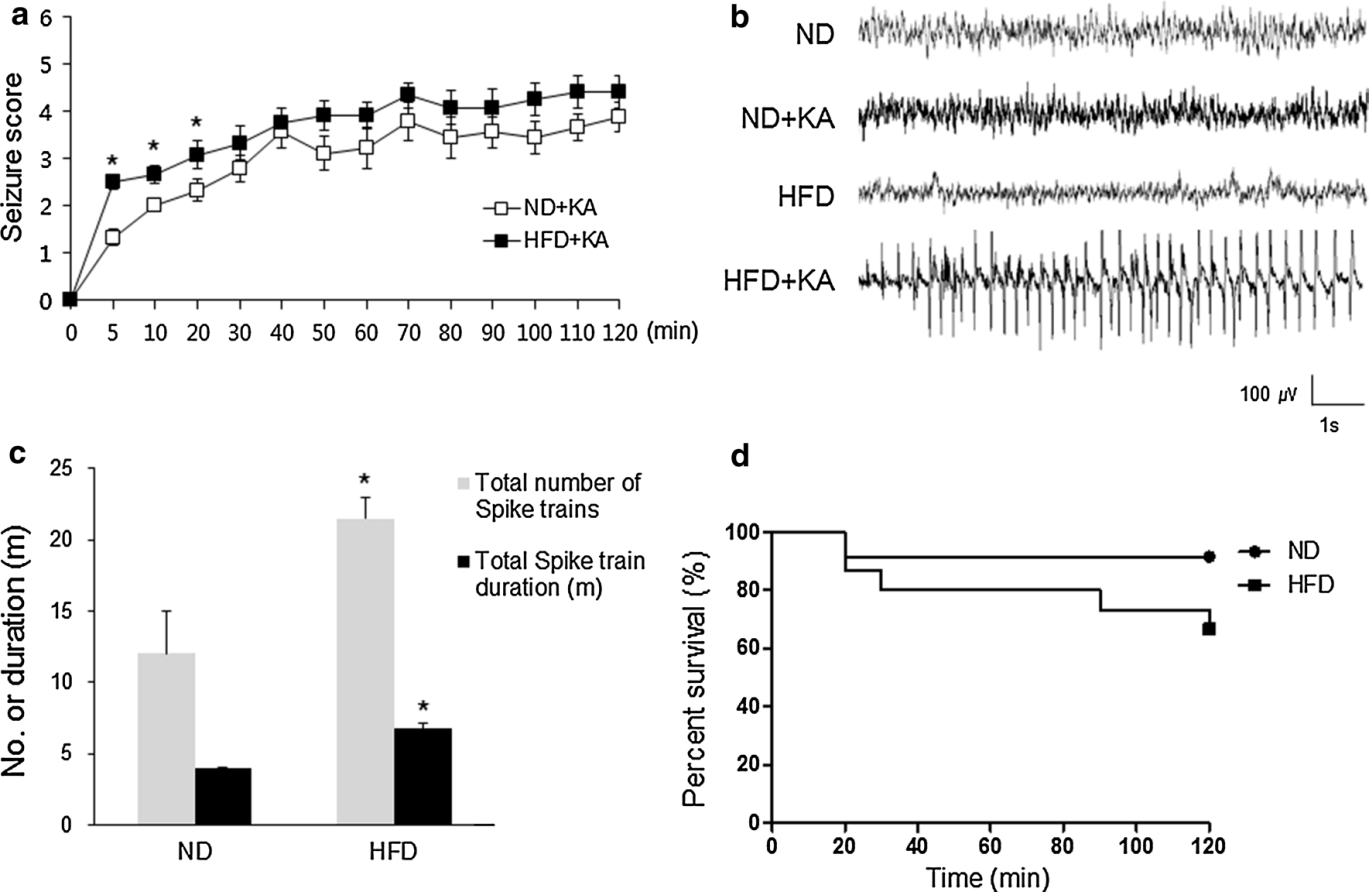
Effects of a HFD on KA-induced seizure activity and mortality. **a** Seizure behavior scores for ND + KA and HFD + KA mice. Scores were recorded every 5 or 10 min. **b** EEG recording within 5 min of a spontaneous seizure in ND + KA and HFD + KA mice. **c** Quantification of EEG recordings during 4 h after KA injection in ND + KA and HFD + KA mice (*n* = 3 mice per group). **d** Percentage of ND + KA and HFD + KA mice that survived for 24 h after KA injection. Data are shown as mean ± SEM. *p < 0.05 for HFD + KA mice versus ND + KA mice

We next examined whether a HFD affects KA-induced seizure severity. HFD mice showed more severe seizure behaviors than ND mice, showing stretching of the body, tail straightening, and bulging eyes during the first 5 min after KA injection (Fig. 1a). To further evaluate seizure activity, we performed electroencephalographic (EEG) recordings. HFD mice showed a characteristic pattern of more spontaneous seizure activity within 5 min after KA treatment (Fig. 1b), and HFD + KA mice exhibited a higher number and longer duration of seizure spike trains than ND + KA mice (Fig. 1c). HFD + KA mice also displayed lower survival rates than ND + KA mice (Fig. 1d).

### Effects of a HFD on KA-induced hippocampal cell death

**Fig. 2:**
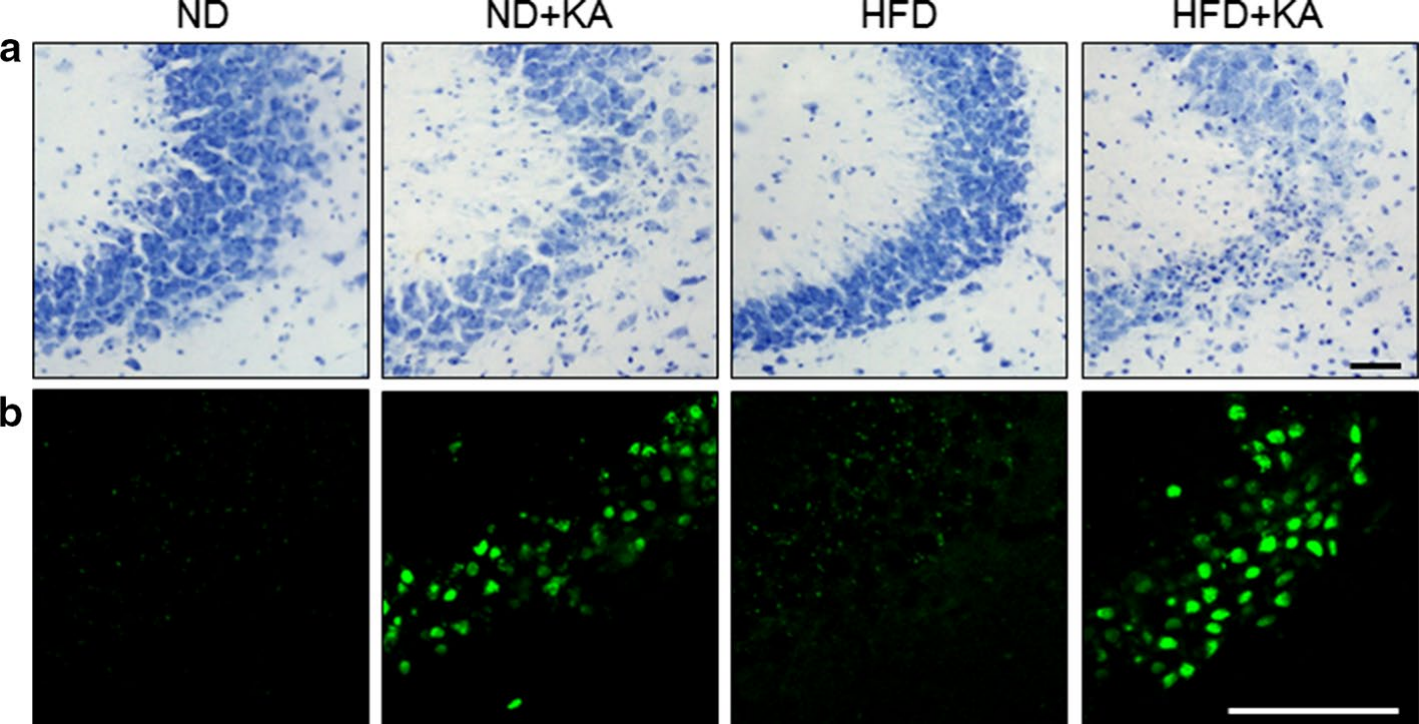
Effects of a HFD on KA-induced hippocampal cell death and calcium overload. Representative microphotographs of* cresyl violet* staining (**a**) and TUNEL staining (**b**) in the CA3 region of ND, ND + KA, HFD, and HFD + KA mice. *Scale* *bar* 50 μm

Upon examination of cresyl violet-stained sections, we found more pyknotic nuclei, typically found in apoptotic cells, in the CA3 region of ND + KA and HFD + KA mice (Fig. 2a). To confirm that KA induced neuronal death, we performed Terminal deoxynucleotidyl transferase dUTP nick end labeling (TUNEL) staining (Fig. 2b). Both ND + KA and HFD + KA mice showed a dramatic increase in TUNEL-positive cells in the hippocampus. HFD + KA mice (22.4 ± 1.5) showed more TUNEL-positive cells than ND + KA mice (19.3 ± 0.8, P < 0.05).

**Fig. 3:**
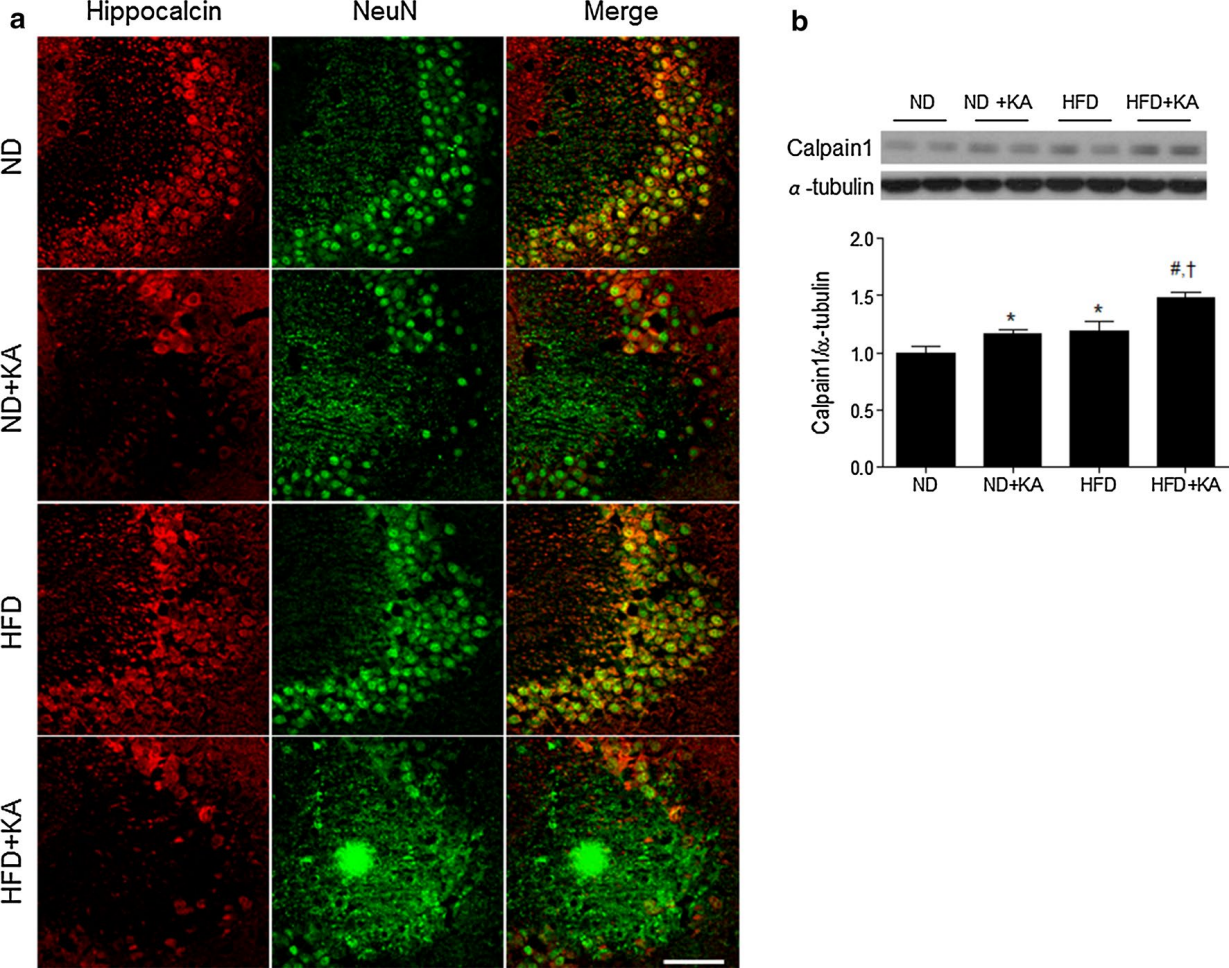
Effects of a HFD on hippocalcin and calpain1 expression KA-treated hippocampus. **a** Representative microphotographs of double immunofluorescence of hippocalcin and NeuN in the CA3 region of ND, ND + KA, HFD, and HFD + KA mice. *Scale bar* 50 μm. **b** Western blot analysis of calpain1. Densitometry values for each protein were normalized to α-tubulin and expressed as fold change relative to the ND group. Data are shown as mean ± SEM (n = 4 mice per group). *p < 0.05 versus ND. ^#^ *p* < 0.05 versus HFD. ^†^ *p* < 0.05 versus ND + KA

Together, these results indicate that HFD exacerbates KA-induced neuronal death. To investigate whether KA affects calcium-mediated cell death in HFD-fed mice, we performed immunohistochemistry with hippocalcin and western blot analysis with calpain1, a calcium-dependent protease. Double immunostaining showed a lack of hippocalcin-positive calcium-buffering neurons (NeuN-positive) in the hippocampus of both ND + KA and HFD + KA mice (Fig. 3a). We found that calpain1 expression was increased in ND + KA-and HFD mice compared with ND mice, and a HFD augmented the KA-induced increase in calpain1 expression (Fig. 3b).

### Effects of a HFD on KA-induced neuroinflammation

**Fig. 4:**
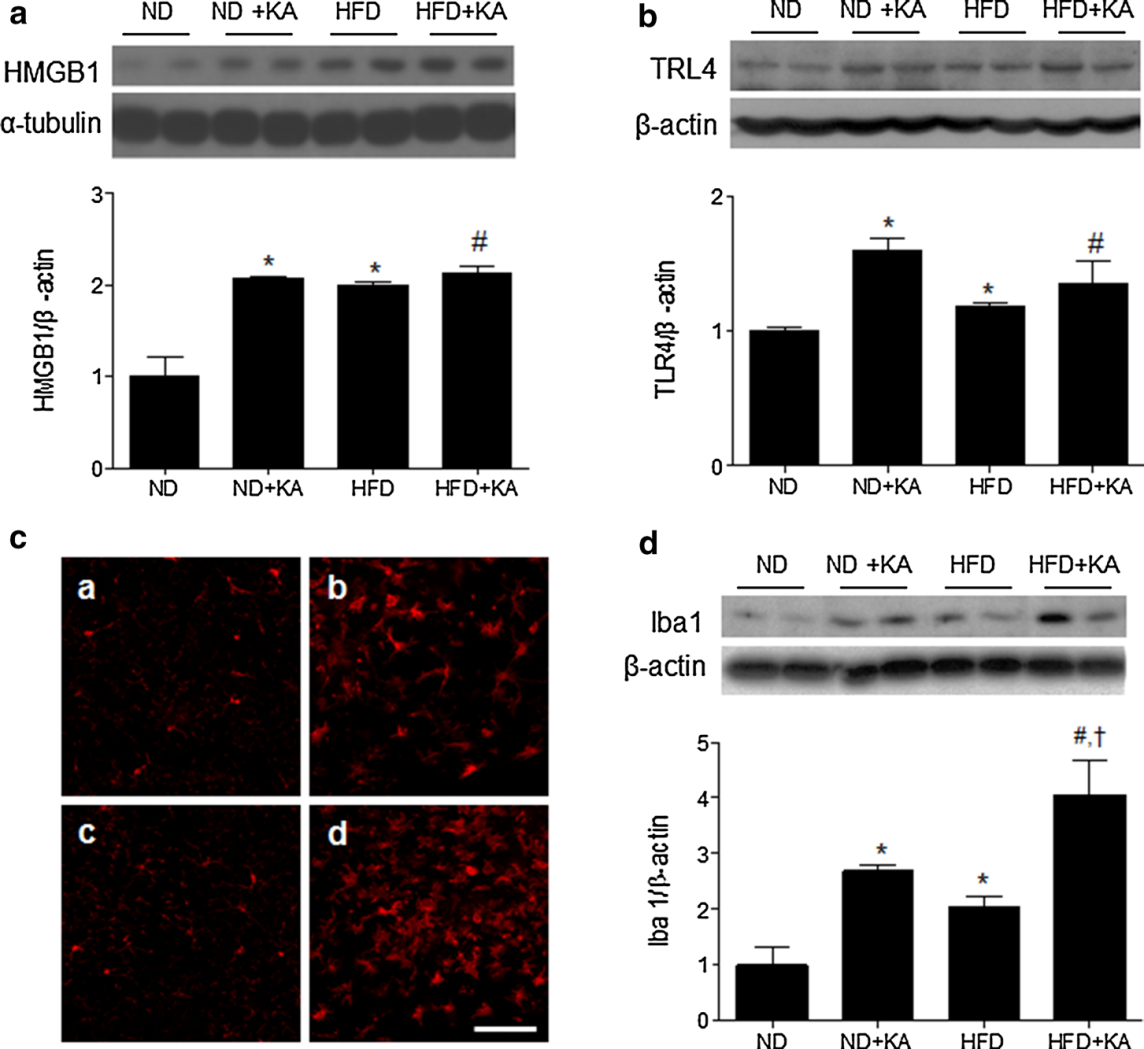
Effects of a HFD on KA-induced neuroinflammation. Western blot analysis of HMGB1 (**a**) and TRL4 (**b**). Densitometry values for each protein were normalized to β-actin and expressed as fold change relative to the ND group. **c** Representative immunofluorescence images of iba-1 in the CA3 region after KA treatment. *Scale bar* 50 µm. **a** ND; **b** ND + KA; **c** HFD; **d** HFD + KA. **d** Western blot analysis of hippocampal iba-1 levels after KA treatment. Densitometry values for iba-1 were normalized to β-actin and expressed as fold change relative to the ND group. Data are shown as mean ± SEM (n = 4 mice per group). *p < 0.05 versus ND. ^#^ *p* < 0.05 versus HFD. ^†^ *p* < 0.05 versus ND + KA

Previous studies show that KA treatment induces neuroinflammation and microglial activation through high mobility group box 1 (HMGB1) and its receptor, toll-like receptor 4 (TLR4) [18, 19]. We confirmed that KA treatment increased hippocampal HMGB1 and TLR4 expression (Fig. 4a, b). We also showed that a HFD in the absence of KA treatment also increased hippocampal HMGB1 and TLR4 expression. Then, we examined iba-1 immunoreactivity and found that the increased labeling in the CA3 region of ND + KA mice and even greater labeling in the CA3 region of HFD + KA mice (Fig. 4c). Consistent with these results, western blot analysis showed that a HFD significantly increased the KA-induced expression of iba-1 protein (Fig. 4d). We also observed an upregulation of hippocampal cyclooxygenase-2 (COX-2) expression in ND + KA, HFD, and HFD + KA mice compared with ND-fed mice (Additional file 4: Fig. S3A). However, there was no significant change in both ND + KA and HFD + KA mice. Immunohistochemistry revealed intense COX-2-staining in the dentate gyrus (DG) and CA3 regions after KA treatment, particularly in HFD + KA mice (Additional file 4: Fig. S3B). Additionally, we also found inducible nitric oxide synthase (iNOS) expression is increased in the hippocampus of ND + KA, HFD, and HFD + KA mice compared with ND-fed mice (Additional file 4: Fig. S3C).

### Effects of a HFD on KA-induced ER stress and Nrf2/HO-1 defense pathway

The ER response involving protein kinase RNA-like ER kinase (PERK) and its downstream activating transcription factor4 (ATF4) are essential for ER stress-induced apoptosis [20]. To examine whether HFD alters expression of ER stress-induced proteins in the hippocampus after KA treatment, we performed western blot analysis. We found that both KA treatment and a HFD increased PERK and ATF4 expression (Additional file 5: Fig. S4A). These results suggest that HFD contributes to KA-induced neuronal death by increasing ER stress signaling, particularly the PERK-ATF4 pathway. In addition, consistent with an increase in ER stress, we found that both KA treatment and a HFD increased 4-hydroxynonenal (4-HNE) expression (Additional file 5: Fig. S4B).

**Fig. 5:**
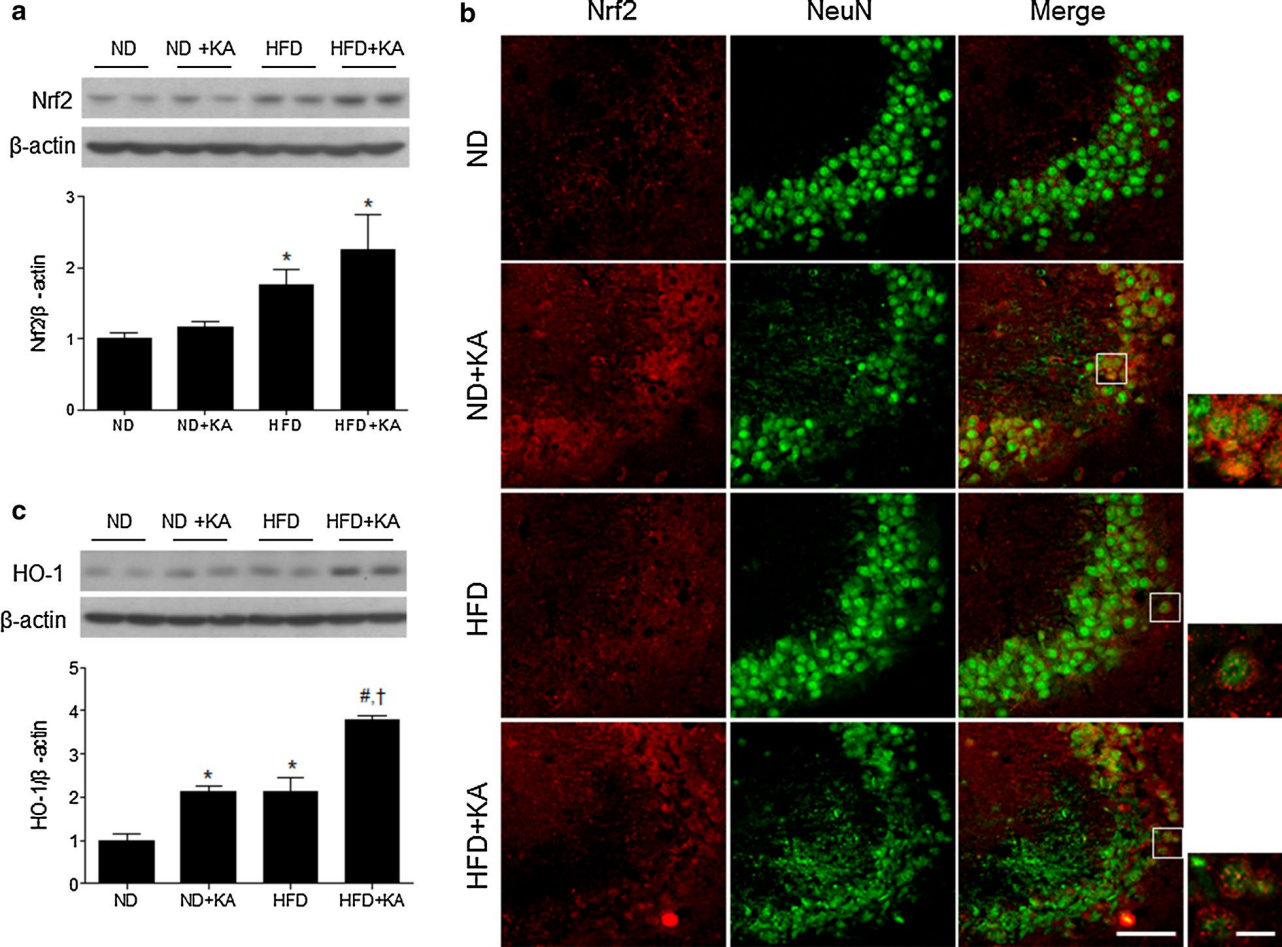
Effects of HFD on KA-induced hippocampal Nrf2 and HO-1 expression. **a** Western blot analysis of Nrf2. **b** Representative immunofluorescence images of Nrf2 (*red*) and NeuN (*green*) in the hippocampus of ND and HFD mice with or without KA treatment. *Scale bar* 50 µm. **c** Western blot analysis of HO-1 in the hippocampus of ND and HFD mice with or without KA treatment. Densitometry values for Nrf2 and HO-1 were normalized to β-actin and expressed as fold change relative to the ND group. Data are shown as mean ± SEM (n = 4 mice per group). *p < 0.05 versus ND. ^#^ *p* < 0.05 versus HFD. ^†^ *p* < 0.05 versus ND + KA

Nrf2 signaling plays an important role in protection against brain injury [21]. Therefore, we examined whether a HFD affects hippocampal Nrf2 expression in response to KA treatment using western blot analysis and double immunofluorescence staining. Western blot analysis showed that Nrf2 expression was increased in HFD and HFD + KA mice compared with ND mice (Fig. 5a). We also observed that KA-treated mice showed a dramatic increase of Nrf2 in the nucleus of NeuN-positive neurons (Fig. 5b). Activation of Nrf2 in response to brain injury may be dependent on the induction of antioxidant response element (ARE)-related genes [22]. Therefore, we assessed hippocampal heme oxygenase-1 (HO-1) and NAD(P)H:quinone oxidoreductase-1 (NQO1) expression using western blot analysis. ND + KA or HFD mice exhibited greater HO-1 and NQO1 expression than ND mice, and a HFD augmented the KA-induced increase in HO-1 and NQO1 expression (Fig. 5c; Additional file 5: Fig. S4C).

## Discussion

HFD-induced chronic inflammation, mainly elicited by microglia in the brain, exacerbates pathologies by increasing production of reactive oxygen species (ROS) and proinflammatory cytokines. Our findings support previous observations that obesity is associated with seizure susceptibility in children and that metabolic dysfunction, including insulin resistance, hepatic steatosis, and macrophage infiltration in adipose tissue, contributes to neuroinflammation and exacerbates KA-induced neuronal death [1–5]. Interestingly, HFD-induced calpain1 expression was augmented by KA treatment and KA-induced hippocampal cell death was more prominent in HFD-fed mice than in ND-fed mice. Thus, we suggest that metabolic dysfunction-induced inflammation may augment KA-induced calcium overload, neuroinflammation, and oxidative stress and thereby contribute to neuronal cell death.

Metabolic dysfunction, such as dyslipidemia, insulin resistance, and hepatic steatosis, can exacerbate seizure-induced brain injury [7, 23], and conversely the use of common anti-epileptic drugs leads to weight gain and metabolic dysfunction [24]. In particular, a high-fat, low-carbohydrate ketogenic diet (KD), which is sometimes used to treat drug-resistant epilepsy, can lead to hepatic steatosis [18, 25]. In a previous study, KD-fed mice showed lower fasting blood glucose and homeostatic model assessment of insulin resistance (HOMA-IR) levels than mice fed standard chow and HFD-fed mice showed higher glucose and HOMA-IR levels than KD-fed mice [26]. Taken together, these findings suggest that KD as a treatment for drug-resistant epilepsy does not affect insulin resistance but induces hepatic steatosis. Thus, obesity paired with anti-epileptic drugs or a KD may not exacerbate seizure-induced neuronal cell death, but chronic HFD-induced insulin resistance may bring other seizure-related outcomes such as neuroinflammation, ER stress, and ROS.

We found that mice fed a HFD diet for 8 weeks exhibited obesity-related phenotypes and insulin resistance. Then, we tested whether HFD-induced obesity exacerbates seizures. Consistent with evidence that KA-induced neurotoxicity is exaggerated in ob/ob mice [9], we found that KA-treated HFD mice showed more severe seizures and greater mortality than KA-treated ND mice. Also, adiponectin deficiency, which is characteristic of obesity and diabetes, increases seizure sensitivity and neuronal loss in the KA-treated hippocampus [5]. Although adiponectin deficiency alone without metabolic changes may not be sufficient to enhance seizure sensitivity, there is a strong positive correlation between seizure severity and insulin resistance in HFD mice [5]. We previously found that intracerebroventricular injection of recombinant adiponectin protects against BBB breakdown and neuronal cell death [8]. In the present study, HFD mice had lower serum adiponectin levels than ND mice. These findings indicate that metabolic disturbances may exacerbate seizure-induced neuronal injury.

We found that KA-treated HFD mice exhibited more neuroinflammation than KA-treated ND mice. Specifically, hippocampal levels of the microglia marker iba-1 were elevated in ND + KA mice and even further elevated in HFD + KA mice. A HFD can cause peripheral and central inflammation and the induction of ROS in rodents [27]. We also found that a HFD increased levels of the inflammatory cytokines IL-6 and TNF-α. As a recent study reports that KA treatment disrupts the BBB [14], we speculate that HFD-induced induction of inflammatory cytokines may exacerbate KA-induced inflammation through a breakdown of the BBB.

Levels of HMGB1 are increased in children with febrile seizures [28], and increased expression of HMGB1 contributes to the generation and perpetuation of seizures in humans or mouse models of epilepsy [21]. Furthermore, antagonists of HMGB1 can decrease seizure recurrence [29]. Consistent with our previous study showing that KA treatment induces microglial activation through HMGB1 [14], we found increased HMGB1 and TLR4 expression in the hippocampus of KA-treated mice with or without HFD and then KA-induced microglial activation was augmented by KA treatment. This data indicate that HFD-induced systemic inflammation may exacerbate KA-induced neuroinflammation through microglial activation.

Oxidative stress is an important factor contributing to excitotoxicity-induced brain injury. ROS enhances glutamate release and expression of specific genes of lipid peroxidation and DNA oxidation resulting in neuronal death [30]. In a previous study, valproic acid, which is commonly used to treat epileptic seizures, reduced the number of TUNEL-, iba-1-, and 4-HNE-positive cells in rats with ischemic injury [31]. Consistently, higher levels of 4-HNE were found in the hippocampus of rats with soman-induced seizures [32], suggesting that increases in lipid peroxidation products might be due to excitotoxicity-induced increases of NO-related reactive radicals in the KA-treated hippocampus. Alternatively, mild oxidative stress induced by a KD may activate an Nrf2-mediated antioxidant response, leading to a decrease in ROS production [33].

In addition to oxidative stress, KA treatment causes a persistent increase in intracellular Ca^2+^ and disruption of ER-mediated Ca^2+^ homeostasis in hippocampal neurons [34]. Our previous study demonstrates that an ER stress inhibitor attenuates KA-induced Ca^2+^ signaling and activation of the PERK-ATF4 pathway 6 h after KA treatment [13]. In the present study, we found that although both a HFD and KA treatment increased hippocampal levels of PERK and ATF4, their expressions in HFD-treated mice were not significantly increased by KA treatment. This data indicate that chronic HFD itself may contribute to ER stress in the brain.

Nrf2 participates in the modulation of microglia under conditions of lipopolysaccharide- or MPTP-induced neuroinflammation [35]. In the present study, we observed increased levels of Nrf2/HO-1 in the KA-treated hippocampus and even greater levels in the hippocampus of HFD + KA-treated mice. In agreement with our results, a previous study showed higher Nrf2 mRNA levels in the cerebral cortex of HFD mice compared with ND mice [26]. We found that in addition to elevated nuclear Nrf2 content, total Nrf2 was also increased in HFD + KA mice. A previous study reports that Nrf2 knockout mice display increased sensitivity to KA-induced seizures [15], suggesting that Nrf2 activation is an important defense against the deleterious outcomes of KA treatment. Similar to Nrf2 expression, we found that KA-induced HO-1 and NQO1 expression was further increased by HFD, suggesting that activation of Nrf2/ARE may be a response to the seizure-induced brain damage. Mostly, both HO-1 and NQO1 are considered as representative antioxidant proteins via Nrf2 signaling. Because mouse NQO1 promoter gene contains an Nrf2-binding consensus regulatory sequence known as ARE [36]. A previous study suggests that inhibitor of HO-1 do not improve neuronal survival and increases mortality in KA-injected rats [37], but pretreatment with the anti-oxidant curcumin attenuates KA-induced neuronal death by controlling HO-1 expression [10]. However, our results suggest that activation of the Nrf2/HO-1/NQO1 pathway may respond to chronic HFD-induced oxidative stress and aggravate brain insults such as ischemia, trauma, and seizures. Thus, we suggest that Nrf2 is involved in regulating the expression of both HO-1 and NQO1 against KA-induced oxidative or inflammatory insults.

## Conclusions

In conclusion, the results of this study suggest that complex mechanisms affected by obesity-induced systemic inflammation, neuroinflammation, ER stress, calcium overload, and oxidative stress may contribute to neuronal death after brain injury.

## Methods

### Animals

Male ICR mice (5 weeks old) were purchased from KOATECH Co. (Pyeongtaek, South Korea) and maintained in the animal facility at Gyeongsang National University. All animal experiments were approved by the institutional review board at Gyeongsang National University and were performed in accordance with the National Institutes of Health guidelines for laboratory animal care. Mice were individually housed with a 12-h light/dark cycle and had free access to food pellets and tap water.

### HFD and seizure induction

Mice were fed with either a normal laboratory chow diet (ND, 2018S; 18 kcal % fat, 58 kcal % carbohydrate, and 24 kcal % protein, Harlan Laboratories, Inc., Indianapolis, IN, USA) or a HFD (D12079B; 60 kcal % fat, 20 kcal % carbohydrate, and 20 kcal % protein, Research Diets, New Brunswick, NJ, USA) ad libitum for 8 weeks and then received an intraperitoneal injection of 30 mg/kg KA (Abcam, Cambridge, MA, USA) emulsified in 0.9 % normal saline. Mice were divided randomly into four groups: ND (*n* = 14), HFD (*n* = 14), ND and KA injection (ND + KA, *n* = 20), or HFD and KA injection (HFD + KA, *n* = 20). Mice were monitored for seizure behavior for 2 h after KA injection. Seizure severity was assessed by a 6-point scale (I–VI) as previously described [38].

### EEG recording

For EEG recordings of KA-induced seizures, we used a small animal radio telemetry system (Data Sciences International, St. Paul, MN, USA) to monitor brain electrical activity in freely mobile ND + KA or HFD + KA mice. EEG recordings were performed after surgical implantation of telemetric transmitters (TA10EA-F20; Data Sciences International) using procedures adapted from those used in a previous study with similar equipment [39]. Prior to surgery, mice were anesthetized with zoletil (0.5 mg/kg, Virbac Laboratories, Carros, France). EEG activity was monitored for 6 h as previously described [14]. To distinguish between abnormal interictal spikes and physiologically occurring spikes in epidural one-channel EEG, we manually counted only spikes that were followed by a wave.

### GTT and ITT

For the GTT, mice were fasted overnight (16 h) and then received an intraperitoneal injection of d-glucose (2 g/kg; Sigma-Aldrich, St. Louis, MO, USA). Blood samples were then collected from the tail vein. For the ITT, mice were given a intraperitoneal injection of insulin (0.75 U/kg, Humulin-R; Eli Lilly, Indianapolis, IN, USA), and blood glucose was measured using an Accu-Chek glucometer (Roche Diagnostics GmbH, Mannheim, Germany).

### Tissue preparation and staining

For tissue analysis, mice were anesthetized with zoletil (5 mg/kg, Virbac Laboratories) and then perfused transcardially with heparinized saline followed by 4 % paraformaldehyde in 0.1 M phosphate-buffered saline (PBS). After post-fixation in the same fixative for 6 h, brains were sequentially immersed in PBS containing 15 % and then 30 % sucrose at 4 °C until they were completely submerged. Brains were cut into 40-μm thick coronal sections and stained with cresyl violet. Livers and epididymal fat pads were processed for paraffin embedding, sectioned (5 µm), and stained with hematoxylin and eosin (H&E). Sections were visualized under a BX51 light microscope (Olympus, Tokyo, Japan), and digital images were captured.

### Hepatic TG colorimetric assay

Frozen livers were freshly homogenized, and the supernatants were used to determine TG levels. TG concentration (*n* = 6 mice per group) was measured using a TG colorimetric assay kit (Cayman Chemical Company, Ann Arbor, MI, USA).

### Measurement of serum metabolic parameters

Mice were intramuscularly anesthetized with zoletil (5 mg/kg; Virbac Laboratories), and blood samples were extracted transcardially and allowed to clot for 2 h at room temperature. After centrifugation, serum samples were stored at −80 °C until analysis. Serum AST, ALT, FFA, total cholesterol, and TG levels were determined using enzymatic colorimetric assays (Green Cross Reference Laboratory, Yongin-si, South Korea). Serum insulin and adiponectin concentrations in ND and HFD mice (*n* = 8–10 mice per group) were measured using mouse insulin (Shibayagi Co., Gunma, Japan) and adiponectin (Shibayagi) enzyme-linked immunosorbent assay (ELISA) kits, respectively. Serum IL-6 and TNF-α concentrations (*n* = 8–10 mice per group) were measured using mouse IL-6 and TNF-α ELISA kits (R&D Systems, MN, USA), respectively.

### TUNEL staining

TUNEL analysis was performed to measure the degree of apoptosis in tissue using an in situ cell death detection kit (Roche Molecular Biochemicals, Mannheim, Germany) according to the manufacturer’s protocol. Stained brain sections were visualized with a confocal microscope (FV-1000, Olympus), and digital images were captured. For each treatment group, TUNEL-positive cells were manually counted in the CA3 region (50 × 50 µm) in three sections (n = 3 mice per group). The cells were counted by observers blinded to the treatment conditions using 20× objectives.

### Immunofluorescence

For double immunofluorescence staining, frozen free-floating brain sections were incubated with primary antibodies (Additional file 6: Table S2); rabbit anti-hippocalcin, mouse anti-NeuN, rabbit anti-Nrf2 at 4 °C for 2 days. After washing three times with PBS, sections were incubated with Alexa Fluor 488- or 594-conjugated donkey anti-rabbit or anti-mouse antibody (Invitrogen, Carlsbad, CA, USA). Fluorescence was visualized with a confocal microscope (FV-1000, Olympus), and digital images were captured.

### Immunohistochemistry

Frozen free-floating brain sections were incubated with rabbit anti-COX-2 (Additional file 6: Table S2) overnight at 4 °C. After three washes with PBS, sections were incubated for 1 h at room temperature with biotinylated secondary antibody. After washing three times with PBS, sections were incubated in avidin–biotin-peroxidase complex solution (Vector Laboratories, Burlingame, CA, USA). Sections were developed with 0.025 % diaminobenzidine (Sigma) containing 0.025 % H_2_O_2_, mounted on gelatin-coated slides, warm-dried, dehydrated through graded alcohols, cleared in xylene, and finally coverslipped with Permount (Sigma). Stained sections were visualized with a BX51 light microscope (Olympus), and digital images were captured.

### Western blot analysis

After anesthesia with zoletil (5 mg/kg; Virbac Laboratories), brains were quickly removed from skulls, and both hippocampi were dissected and frozen. Hippocampal whole cellular extracts and nuclear fractions were prepared as previously described [38]. Frozen hippocampi were individually transferred to sterile 1.5-ml microcentrifuge tubes containing 200 µl lysis buffer (15 mM HEPES, pH 7.9, 0.25 M sucrose, 60 mM KCl, 10 mM NaCl, 1 mM EGTA, 1 mM PMSF, and 2 mM NaF). Homogenized tissue was incubated for 10 min on ice and then sonicated. Samples were then centrifuged at 4 °C for 30 min at 12,000 rpm. The supernatants were transferred to clean vials and stored at −80 °C. The protein concentration of each lysate was determined using a bicinchoninic acid kit (Pierce, Rockford, IL, USA) according to the manufacturer’s protocol, using bovine serum albumin as a standard. Equal amounts of protein (30 μg) were separated by SDS-PAGE and transferred to nitrocellulose membranes. The membranes were washed in Tris-buffered saline containing 0.5 % Tween-20 and incubated with the following primary antibodies (Additional file 6: Table S2): iba-1, HMGB1, TLR4, 4-HNE, PERK, ATF4, HO-1, NQO1, COX-2, or iNOS. Samples were then incubated with their corresponding secondary antibodies. The enhanced chemiluminescence western blot analysis system (Amersham Pharmacia Biotech, Piscataway, NJ, USA) was used for detection. To normalize protein levels, α-tubulin or β-actin was used as an internal control.

### Statistical analysis

Differences among experimental groups were determined using one-way analysis of variance (ANOVA) followed by post hoc analysis with Student–Newman–Keuls tests. Student’s *t*-tests were used for comparisons between two groups. Values are expressed as mean ± standard error of the mean (SEM). Statistical significance was set at *p* < 0.05.

## Additional files

10.1186/s12868-015-0202-2 Effects of a HFD on obesity-related phenotypes. Body weight (A) and external phenotype (B); liver and epididymal fat pad weight (C), gross appearance (D), and representative H&E staining (E) in liver and epididymal fat; and levels of hepatic TG (F) were assessed in mice fed a ND or HFD for 8 weeks. Data are presented as mean ± SEM (n = 8-10 mice per group). *p < 0.05 for HFD mice versus ND mice. Scale bar = 1 cm (B and D), 100 μm (E).
10.1186/s12868-015-0202-2 Effects of a HFD on serum metabolic parameters.
10.1186/s12868-015-0202-2 Effects of a HFD on insulin resistance. Fasting blood glucose (A) in mice fed a ND or HFD for 8 weeks. Serum insulin levels (B), glucose levels in ITTs (C) and GTTs (D), adiponectin (E), IL-6 (F), and TNF-α (G) were measured in mice fed a ND or HFD. Data are presented as mean ± SEM (n = 8-10 mice per group). *p < 0.05 for HFD mice versus ND mice.
10.1186/s12868-015-0202-2 Effects of a HFD on KA-induced hippocampal COX-2 and iNOS expression. Western blot and quantification of COX-2 (A) and iNOS (C) in the hippocampus after KA treatment. Densitometry values for each protein were normalized to α-tubulin or β-actin and expressed as fold change relative to the ND group. Data are shown as mean ± SEM (n = 4 mice per group). *p < 0.05 versus ND. #p < 0.05 versus HFD. (B) Representative images of COX-2 immunostaining in the hippocampus of ND and HFD mice with or without KA treatment. Scale bar = 100 µm.
10.1186/s12868-015-0202-2 Effects of HFD on KA-induced ER stress. Western blot analysis of PERK and ATF4 (A), 4-HNE (B), and NQO1 (C) in the hippocampus after KA treatment. Densitometry values for each protein were normalized to α–tubulin or β-actin and expressed as fold change relative to the ND group. Data are shown as mean ± SEM (n = 4 mice per group). *p < 0.05 versus ND. #p < 0.05 versus HFD.
10.1186/s12868-015-0202-2 List of primary antibodies.

## Abbreviations

ALT: alanine aminotransferase
AST: aspartate aminotransferase
ATF4: activating transcription factor 4
BBB: blood–brain barrier
CHOP: C/EBP homology protein
COX-2: cyclooxygenase-2
EEG: electroencephalographic
elF2α: eukaryotic translation initiation factor 2α
FFA: free fatty acid
ER: endoplasmic reticulum
GTT: glucose tolerance test
4-HNE: 4-hydroxynonenal
HFD: high-fat diet
HMGB1: high mobility group box 1
HO-1: heme oxygenase-1
HOMA-IR: homeostasis model assessment-estimated insulin resistance
iNOS: inducible nitric oxide synthase
IL-6: interleukin-6
ITT: insulin tolerance test
KA: kainic acid
KD: ketogenic diet
NF-κb: nuclear factor-kappaB
Nrf2: nuclear factor erythroid 2-related factor 2
NQO1: NAD(P)H:quinone oxidoreductase-1
PERK: ER response involving protein kinase RNA-like ER kinase
TG: triglyceride
TLR4: toll-like receptor 4
TUNEL: terminal deoxynucleotidyl transferase dUTP nick end labeling
TNF-α: tumor necrosis factor-α
