# Erratum to: High-fat diet-induced obesity exacerbates kainic acid-induced hippocampal cell death

**DOI:** 10.1186/s12868-017-0338-3

**Published:** 2017-01-23

**Authors:** Dong Ho Kang, Rok Won Heo, Chin-ok Yi, Hwajin Kim, Chang Hwa Choi, Gu Seob Roh

**Affiliations:** 1 Department of Neurosurgery, Institute of Health Sciences, Gyeongsang National University School of Medicine, 15, Jinju-daero 816 Beon-gil, Jinju-si, Gyeongnam Republic of Korea; 2 Department of Anatomy and Convergence Medical Science, Institute of Health Sciences, Gyeongsang National University School of Medicine, 15, Jinju-daero 816 Beon-gil, Jinju-si, Gyeongnam Republic of Korea; 3 Department of Neurosurgery, Pusan National University School of Medicine, 179 Gudeok-ro, Seo-gu, Busan, Republic of Korea

## Erratum to: BMC Neurosci (2015) 16:72 DOI 10.1186/s12868-015-0202-2

In this version of this article that was originally published [1], there is incorrect funding information.

The funding information is currently:

This study was supported by a Grant of the Korean Health Technology R&D Project, Ministry of Health & Welfare, Republic of Korea (A111436) and the Basic Science Research Program through the National Research Foundation (NRF) of Korea (Nos. 2014-0420, 2014R1A2A1A11049588 and 2015R1A5A2008833).

However, the number 2014-0420 is incorrect and should be 2014R1A1A1003211.
