# Supplementary figures and images for: High-fat diet-induced obesity exacerbates kainic acid-induced hippocampal cell death
Source: BMC Neurosci. 2015 Oct 30;16:72. doi: 10.1186/s12868-015-0202-2 (PMC4628384; doi:10.1186/s12868-015-0202-2)

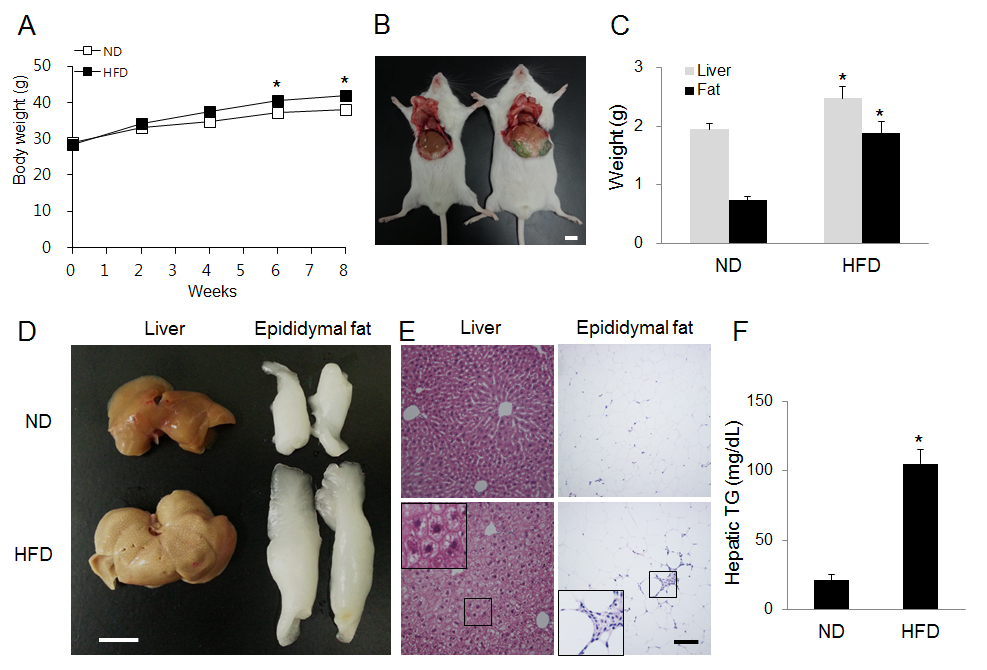

Supplement: Supplementary file 1 — 10.1186/s12868-015-0202-2 Effects of a HFD on obesity-related phenotypes. Body weight (A) and external phenotype (B); liver and epididymal fat pad weight (C), gross appearance (D), and representative H&E staining (E) in liver and epididymal fat; and levels of hepatic TG (F) were assessed in mice fed a ND or HFD for 8 weeks. Data are presented as mean ± SEM (n = 8-10 mice per group). *p < 0.05 for HFD mice versus ND mice. Scale bar = 1 cm (B and D), 100 μm (E). [file 12868_2015_202_MOESM1_ESM.tif]

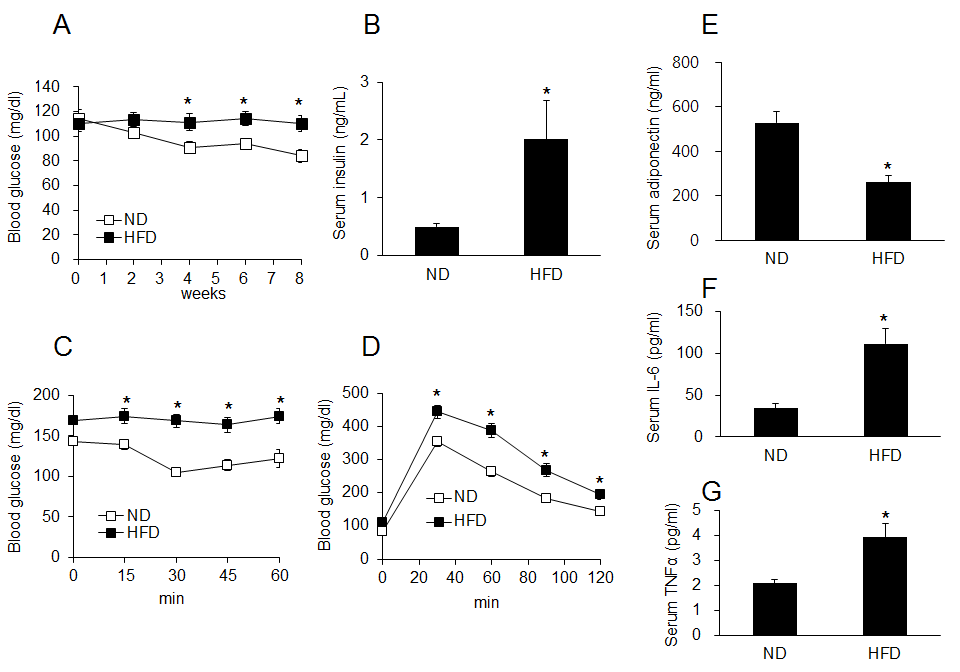

Supplement: Supplementary file 3 — 10.1186/s12868-015-0202-2 Effects of a HFD on insulin resistance. Fasting blood glucose (A) in mice fed a ND or HFD for 8 weeks. Serum insulin levels (B), glucose levels in ITTs (C) and GTTs (D), adiponectin (E), IL-6 (F), and TNF-α (G) were measured in mice fed a ND or HFD. Data are presented as mean ± SEM (n = 8-10 mice per group). *p < 0.05 for HFD mice versus ND mice. [file 12868_2015_202_MOESM3_ESM.tif]

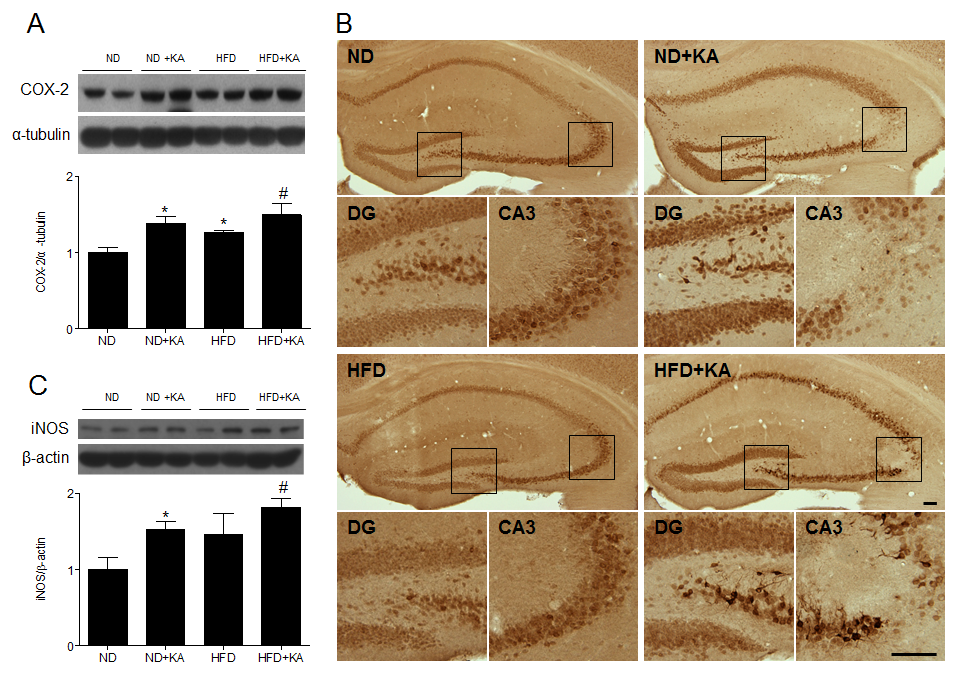

Supplement: Supplementary file 4 — 10.1186/s12868-015-0202-2 Effects of a HFD on KA-induced hippocampal COX-2 and iNOS expression. Western blot and quantification of COX-2 (A) and iNOS (C) in the hippocampus after KA treatment. Densitometry values for each protein were normalized to α-tubulin or β-actin and expressed as fold change relative to the ND group. Data are shown as mean ± SEM (n = 4 mice per group). *p < 0.05 versus ND. #p < 0.05 versus HFD. (B) Representative images of COX-2 immunostaining in the hippocampus of ND and HFD mice with or without KA treatment. Scale bar = 100 µm. [file 12868_2015_202_MOESM4_ESM.tif]

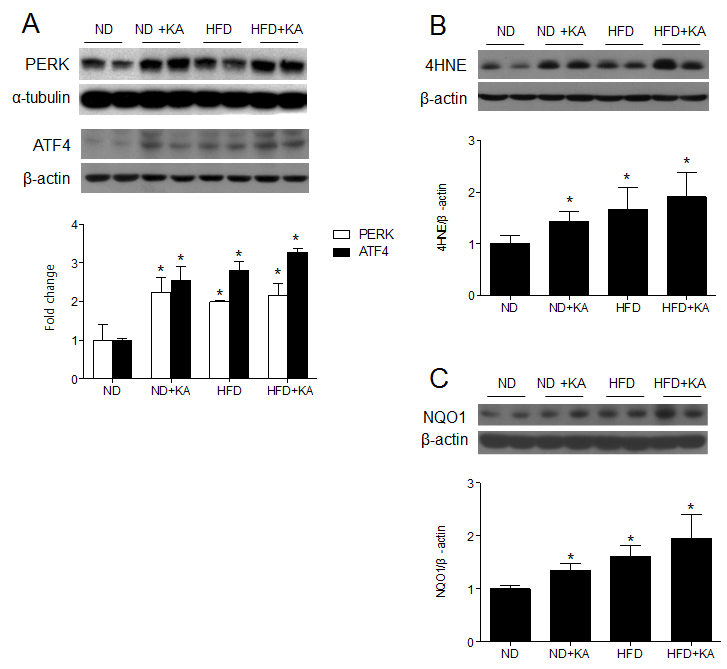

Supplement: Supplementary file 5 — 10.1186/s12868-015-0202-2 Effects of HFD on KA-induced ER stress. Western blot analysis of PERK and ATF4 (A), 4-HNE (B), and NQO1 (C) in the hippocampus after KA treatment. Densitometry values for each protein were normalized to α–tubulin or β-actin and expressed as fold change relative to the ND group. Data are shown as mean ± SEM (n = 4 mice per group). *p < 0.05 versus ND. #p < 0.05 versus HFD. [file 12868_2015_202_MOESM5_ESM.tif]
