# Supplementary material for: High-fat diet-induced obesity exacerbates kainic acid-induced hippocampal cell death
Source: BMC Neurosci. 2015 Oct 30;16:72. doi: 10.1186/s12868-015-0202-2 (PMC4628384; doi:10.1186/s12868-015-0202-2)
Supplement: Supplementary file 2 — 10.1186/s12868-015-0202-2 Effects of a HFD on serum metabolic parameters. [file 12868_2015_202_MOESM2_ESM.docx]

|  | **FFA (μEq/L)** | **AST (U/L)** | **ALT (U/L)** | **T. Chol. (mg/dL)** | **TG (mg/dL)** |
| --- | --- | --- | --- | --- | --- |
| ND (n=8) | 1495.75 ± 113.78 | 84.61 ± 10.06 | 33.83 ± 4.23 | 137.28 ± 7.22 | 115.50 ± 8.55 |
| HFD (n=10) | 1712.40 ± 102.15* | 149.93 ± 49.29* | 91.77 ± 41.59* | 204.20 ± 11.51* | 174.50 ± 19.52* |

Supplementary Table 1. Effects of a HFD on serum metabolic parameters
