# Supplementary material for: High-fat diet-induced obesity exacerbates kainic acid-induced hippocampal cell death
Source: BMC Neurosci. 2015 Oct 30;16:72. doi: 10.1186/s12868-015-0202-2 (PMC4628384; doi:10.1186/s12868-015-0202-2)
Supplement: Supplementary file 6 — 10.1186/s12868-015-0202-2 List of primary antibodies. [file 12868_2015_202_MOESM6_ESM.docx]

**Supplementary Table 2. List of primary antibodies**

| **Antibody** | **Company** | **Catalog No.** | **Dilution** | **Applications** | **Source** |
| --- | --- | --- | --- | --- | --- |
| Iba1 | Wako | 016-20001  019-19741 | 1:1000  1:1000 | WB  IF | Rabbit  Rabbit |
| PERK | Cell signaling | 3192 | 1:1000 | WB | Rabbit |
| ATF4 | Santa Cruz Biotech | sc-200 | 1:1000 | WB | Rabbit |
| HBGB1 | Abcam | ab18256 | 1:1000 | WB | Rabbit |
| TLR4 | Santa Cruz Biotech | sc16240 | 1:1000 | WB | Rabbit |
| NeuN | Millipore | MAB377 | 1:500 | IF | Mouse |
| COX-2 | Cayman chemical | 160126 | 1:1000,1:200 | WB, IHC | Rabbit |
| iNOS | Santa Cruz Biotech | sc-650 | 1:1000 | WB | Rabbit |
| 4-HNE | Abcam | ab48506 | 1:1000 | WB | Rabbit |
| Nrf2 | Santa Cruz Biotech | sc-722 | 1:1000, 1:100 | WB, IF | Rabbit |
| HO-1 | Enzo life science | ADI-SPA-895 | 1:1000, 1:100 | WB, IF | Mouse |
| NQO1 | Abcam | ab34713 | 1:1000 | WB | Rabbit |
| Hippocalcin | Abcam | ab24560 | 1:100 | IF | Rabbit |
| β-actin | Sigma | A5441 | 1:30000 | WB | Mouse |
| α-tubulin | Sigma | T5168 | 1:50000 | WB | Mouse |

WB, western blot; IF, immunofluorescence; IHC, immunohistochemistry
